# A small molecule regulator of tissue transglutaminase conformation inhibits the malignant phenotype of cancer cells

**DOI:** 10.18632/oncotarget.26193

**Published:** 2018-09-28

**Authors:** William P. Katt, Nicolas J. Blobel, Svetlana Komarova, Marc A. Antonyak, Ichiro Nakano, Richard A. Cerione

**Affiliations:** ^1^ Department of Molecular Medicine, Cornell University, Ithaca, NY, USA; ^2^ Department of Neurosurgery, University of Alabama at Birmingham, Birmingham, AL, USA; ^3^ Department of Chemistry and Chemical Biology, Cornell University, Ithaca, NY, USA

**Keywords:** transglutaminase, glioblastoma, signaling, glioma stem cells, cancer

## Abstract

The protein crosslinking enzyme tissue transglutaminase (tTG) is an acyltransferase which catalyzes transamidation reactions between two proteins, or between a protein and a polyamine. It is frequently overexpressed in several different types of human cancer cells, where it has been shown to contribute to their growth, survival, and invasiveness. tTG is capable of adopting two distinct conformational states: a protein crosslinking active (“open”) state, and a GTP-bound, crosslinking inactive (“closed”) state. We have previously shown that the ectopic expression of mutant forms of tTG, which constitutively adopt the open conformation, are toxic to cells. This raises the possibility that strategies directed toward causing tTG to maintain an open state could potentially provide a therapeutic benefit for cancers in which tTG is highly expressed. Here, we report the identification of a small molecule, TTGM 5826, which stabilizes the open conformation of tTG. Treatment of breast and brain cancer cell lines, as well as glioma stem cells, with this molecule broadly inhibits their transformed phenotypes. Thus, TTGM 5826 represents the lead compound for a new class of small molecules that promote the toxicity of cancer cells by stabilizing the open state of tTG.

## INTRODUCTION

Protein-glutamine γ-glutamyltransferase 2, more commonly referred to as tissue transglutaminase or type-2 transglutaminase (tTG, EC = 2.3.2.13), is a member of the transglutaminase family of proteins. These proteins catalyze the crosslinking of a variety of substrates, in which a glutamine residue of one polypeptide chain is covalently linked to either the lysine of another peptide sequence, or to a non-peptidic amine-bearing small molecule, resulting in the formation of an amide bond and the release of ammonia [1].

tTG has been implicated in a number of aspects of cancer progression [1–5]. For example, it was shown to play an important role in growth factor-stimulated cancer cell migration. Specifically, epidermal growth factor (EGF) treatment of HeLa cervical carcinoma cells caused tTG to localize to leading edges where it catalyzed protein crosslinking events necessary for EGF-stimulated cell motility [6]. Recent evidence has also revealed an interesting connection between tTG and the maintenance of cellular pH, as it was shown that the inhibition of tTG crosslinking activity in highly aggressive cancer cells caused a decrease in extracellular pH and resulted in growth inhibition and increased apoptosis [7]. Moreover, a good deal of effort has been directed toward examining the roles played by tTG when it is released by cancer cells into the extracellular environment via non-classical secretory vesicles called microvesicles. The protein crosslinking activity of the microvesicle-associated tTG was shown to promote the ability of these vesicles to activate signaling pathways in target cells that increased their growth and survival [8, 9].

**Figure 1:**
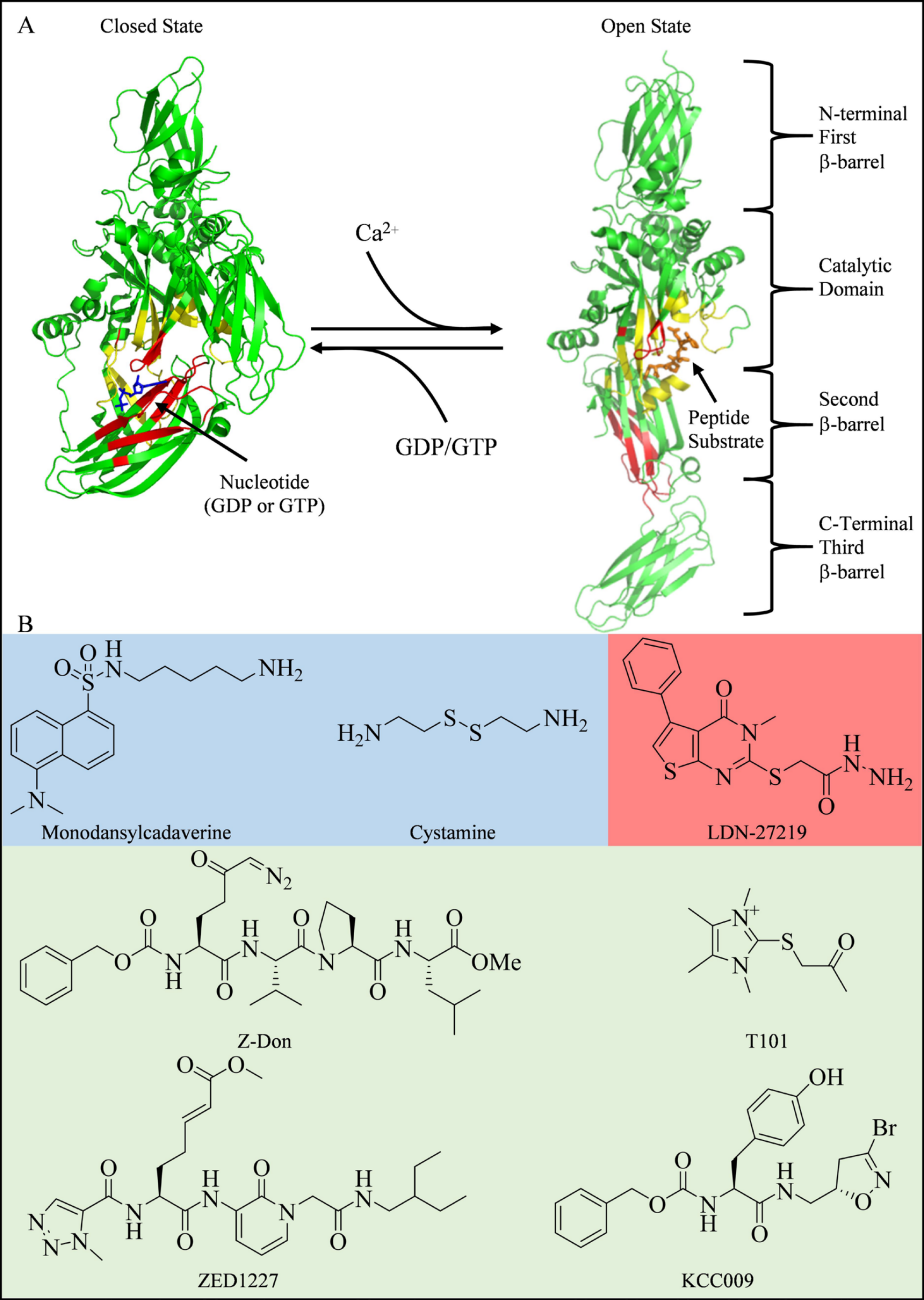
Structures of tTG and some common tTG inhibitors (**A**) tTG adopts two distinct conformational states. In each tTG structure, the nucleotide-binding sites are in red, and the crosslinking active sites are in yellow. On the left is the closed state of tTG (pdb ID 1KV3). The bound nucleotide is in blue. Upon addition of Ca^2+^, tTG is converted to the open state (pdb ID 2Q3Z, right). A covalently bound peptidomimetic inhibitor is shown in orange. The open state can be converted to the closed state via addition of guanine nucleotide. (**B**) Inhibitors of tTG fall into three major categories: 1) alternate substrates, such as monodansylcadaverine [36] and cystamine [37] (shaded blue), which replace the intended glutamine donor substrate, 2) reversible inhibitors such as LDN-27219 [38] (shaded red), which directly competes with nucleotide for binding, or 3) irreversible inhibitors, such as T101 [43], Z-Don [44], ZED1227 [34], and KCC009 [45] (shaded green), which directly compete with crosslinking substrates, by forming a covalent bond with the active site cysteine residue.

tTG is capable of guanine nucleotide-binding and GTP hydrolytic activity. The binding of guanine nucleotides to tTG inhibits its enzymatic protein crosslinking activity. tTG is comprised of four domains: an N-terminal β-barrel domain, the crosslinking catalytic domain, and two additional β-barrel domains (Figure 1A) [10, 11]. When bound to GDP or GTP, tTG adopts what is referred to as the closed state conformation (pdb ID 1KV3, Figure 1A, left side). In this state, the binding of nucleotide (the nucleotide binding domain is colored red) causes the two C-terminal β-barrel domains (colored green) to fold over and block substrate access to the catalytic site (colored yellow). However, in the presence of excess Ca^2+^, tTG has a reduced affinity for guanine nucleotides (i.e. GTP or GDP), causing the two C-terminal β-barrel domains to move away from the catalytic domain. This allows substrate access to the crosslinking active site, and represents the open state conformation of tTG (pdb ID 2Q3Z, Figure 1A, right side). Since the cytosolic levels of Ca^2+^ (~100 nM in resting cells) are much lower than the cellular levels of guanine nucleotides (GTP and GDP combined are in the low mM range), it is generally assumed that tTG is bound to GDP or GTP in cells and predominantly exists in the closed state [1, 12–14].

We and others have demonstrated that mutant forms of tTG, which are defective in their ability to bind guanine nucleotides (e.g. the tTG R580K mutant), constitutively adopt the open state. When these mutants were ectopically expressed in cells, they induced cell death [15, 16]. Interestingly, this occurred even if the guanine nucleotide-binding-defective forms of tTG were further mutated such that they were no longer capable of protein crosslinking activity (e.g. tTG R580K/C277V) [15]. In contrast, wildtype tTG, or tTG mutants that are defective in their enzymatic crosslinking activity but retain their ability to bind guanine nucleotides, primarily adopt a closed state conformation and promote cell survival. Taken together, these findings suggest that the ability of tTG to exist in the open state for extended periods of time is detrimental to cell viability [17]. These diametrically opposed effects of the open and closed states of tTG may be due to their ability to selectively bind different proteins. For example, we have shown that tTG, when in the closed state, is able to bind c-Cbl and prevent it from catalyzing the ubiquitylation and lysosomal degradation of the epidermal growth factor receptor (EGFR). As a result, the build-up of EGFRs on the surfaces of cancer cells promotes cell growth and survival [17]. However, the mechanism by which open state tTG elicits cytotoxic effects in cells is still not understood.

tTG expression has been shown to be highly up-regulated in a large number of cancers. This is especially the case in the most aggressive and high grade cancers, such as triple negative breast cancer and glioblastoma [5, 18, 19]. It is also frequently expressed in cancer initiator or stem cells (CSCs), which are thought to be responsible for driving tumor progression [20, 21]. Indeed, tTG has been shown to be important for the proliferation and survival of several different types of CSCs [22–26], including glioma stem cells (GSCs) [27, 28]. We and others have recently demonstrated that many GSCs express tTG and are sensitive to tTG inhibition [5, 25, 29–31]. These findings, combined with the fact that tTG knockout mice are predominantly healthy, make tTG a potentially promising therapeutic target both for differentiated cancer cells and CSCs [32, 33].

Several classes of small-molecule inhibitors have been described that target the protein crosslinking activity of tTG [34, 35]. Some of these are alternative substrate inhibitors such as monodansylcadaverine (MDC) or cystamine (Figure 1B, blue shaded area), while others, such as LDN-27219, block crosslinking activity by binding to the nucleotide binding site and stabilizing tTG in the closed conformation (Figure 1B, red shaded area) [36–38]. Irreversible inhibitors, such as the peptidomimetic Z-Don or the non-peptidic compound T101 (Figure 1B, green shaded area), have also been extensively investigated [34, 39–45]. In general, the alternative substrates are not highly selective, and the peptidomimetic inhibitors tend to show poor cell permeability, while irreversible inhibitors can potentially lead to protein/inhibitor complex immunogenicity and have slow rates of clearance should toxic effects arise [44, 46, 47]. Because of these limitations, no tTG inhibitor has yet been approved for clinical use.

Given that constitutively maintaining tTG in the open state is cytotoxic, we set out to identify cell permeable molecules that stabilize the enzyme in this conformation. Here we report the identification and characterization of one such molecule, TTGM 5826, which not only enables tTG to maintain the open state, but also functions as a competitive inhibitor of tTG-mediated crosslinking activity. Treating cancer cell lines, as well as GSCs, that express tTG with TTGM 5826 induced cell death at concentrations that were not harmful to non-transformed cells. Thus, this compound potentially opens the way to the development of new strategies for targeting cancer cells exhibiting high levels of tTG expression.

## RESULTS

**Figure 2:**
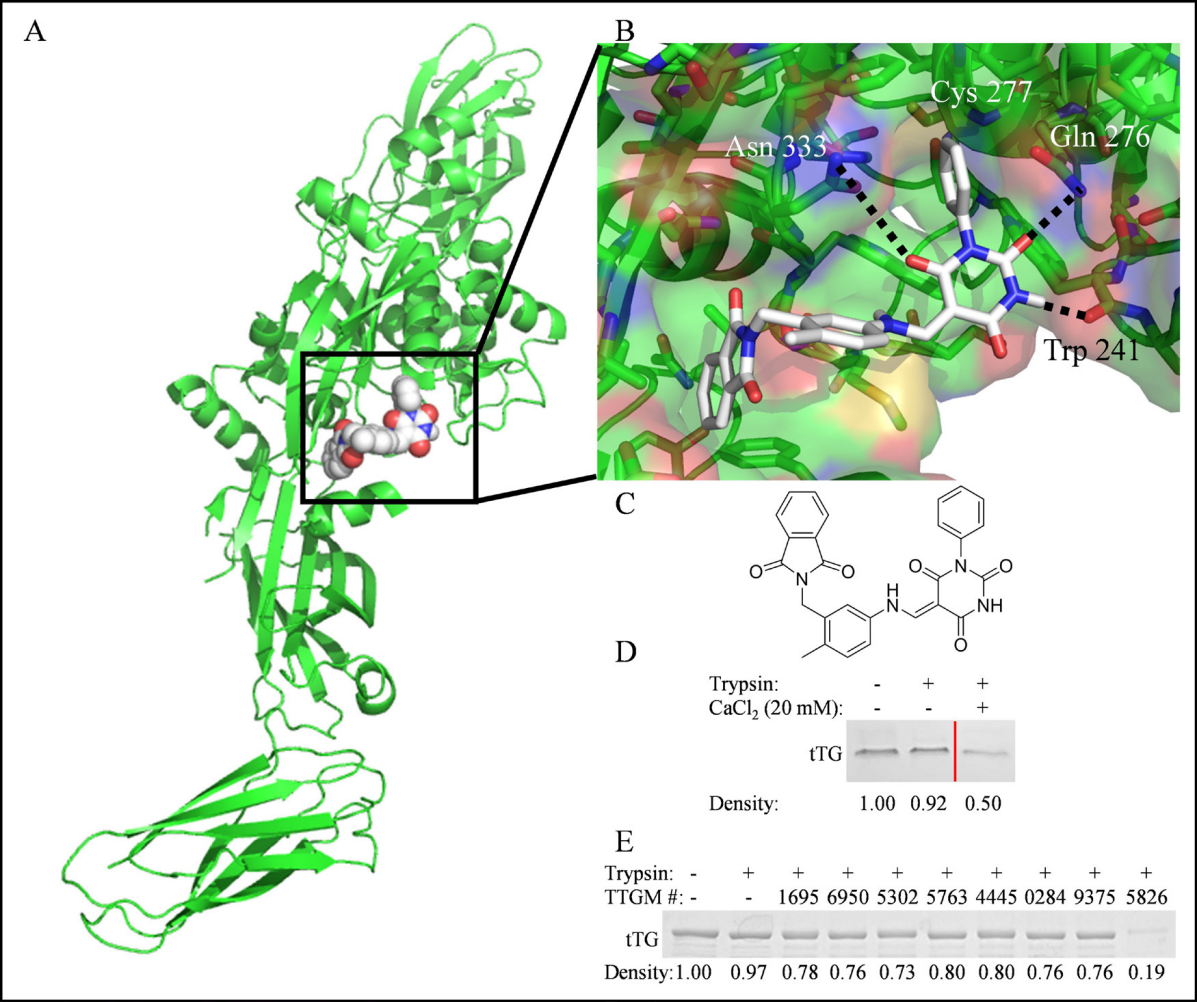
The identification of TTGM 5826 as a potential modulator of tTG conformation (**A**) For screening purposes, molecules were docked to the substrate binding site (boxed) of the open state of tTG (green, pdb ID: 2Q3Z). Shown is TTGM 5826 (space filling, white) docked to tTG. (**B**) Enlarged view of TTGM 5826 (white) docked to the crosslinking active site of tTG (green, pdb ID: 2Q3Z). The phenyl ring of TTGM 5826 projects into a deep pocket formed around the catalytic Cys 277, while the barbiturate ring is predicted to engage in hydrogen bonds with Trp 241, Gln 276, and Asn 333. The flexible linker allows the molecule to wrap around a ‘hump’ in the binding site, while the phthalamide projects into a second deep hydrophobic pocket. (**C**) The chemical structure of TTGM 5826 is composed of four moieties: a phenyl ring (lower left), a barbiturate (upper left), a tolyl ring (upper right), and a phthalamide (lower right). (**D**) tTG was incubated on ice with or without 20 mM CaCl_2_ for 5 minutes, and then each sample was incubated without (lane 1) or with trypsin (lanes 2 & 3) for 3 hours. The samples were resolved by SDS-PAGE and stained with Coomassie blue. Lighter bands indicate that a greater amount of tTG was digested. Lanes of samples where lower concentrations of CaCl_2_ were used were spliced out for clarity, and are indicated by the red line. (**E**) tTG was incubated on ice with the indicated small molecule (TTGM #), or DMSO, for 5 minutes, at which point trypsin was added, as indicated, and the reactions were processed as described in (**C**). TTGM molecules are labeled by the last four digits of their ChemBridge catalog number. Densiometric quantitation was performed with ImageJ. Band densities are reported as fractional density of the trypsin-free control band.

To identify candidate molecules that could potentially stabilize the open state conformation of tTG, we performed a virtual screen to search for molecules that preferentially bound at the crosslinking active site (Figure 2A, boxed region). A library containing ~30,000 small molecules was obtained from the ZINC online database and docked onto tTG using Autodock Vina (Figure 2B). Twenty-four chemically diverse small molecules were identified and selected for further biochemical analysis (Supplementary Figure 1). One of these, TTGM 5826 (Figure 2C), became our lead compound.

### TTGM 5826 stabilizes the open state of tTG

We set out to determine if any of the small molecules identified in our screen were capable of stabilizing the open conformation of tTG using a trypsin digestion assay. For these experiments, we took advantage of earlier findings that showed tTG was more susceptible to trypsin digestion when it is in the extended, open conformation, compared to the more compact, closed conformation [17]. Recombinant tTG was incubated with either DMSO (vehicle control), the different small molecules (1 mM final concentration), or with 20 mM CaCl_2_ (which stabilizes the open conformation) as a positive control. The samples were then treated without or with trypsin prior to being subjected to SDS-PAGE and Coomassie blue staining. Figure 2D shows that tTG, when predominantly in the closed state (i.e. in the absence of Ca^2+^), was resistant to trypsin digestion (compare lanes 1 and 2), whereas, in the presence of 20 mM Ca^2+^, approximately half of the tTG was digested (lane 3). Figure 2E shows the results of a similar assay, where some of the candidate small molecules identified in our screen were examined for their ability to cause tTG to become sensitive to trypsin digestion. Only one compound, TTGM 5826 (last lane), made tTG significantly susceptible to trypsin digestion, suggesting that this compound is capable of inducing and/or stabilizing its open state conformation.

**Figure 3:**
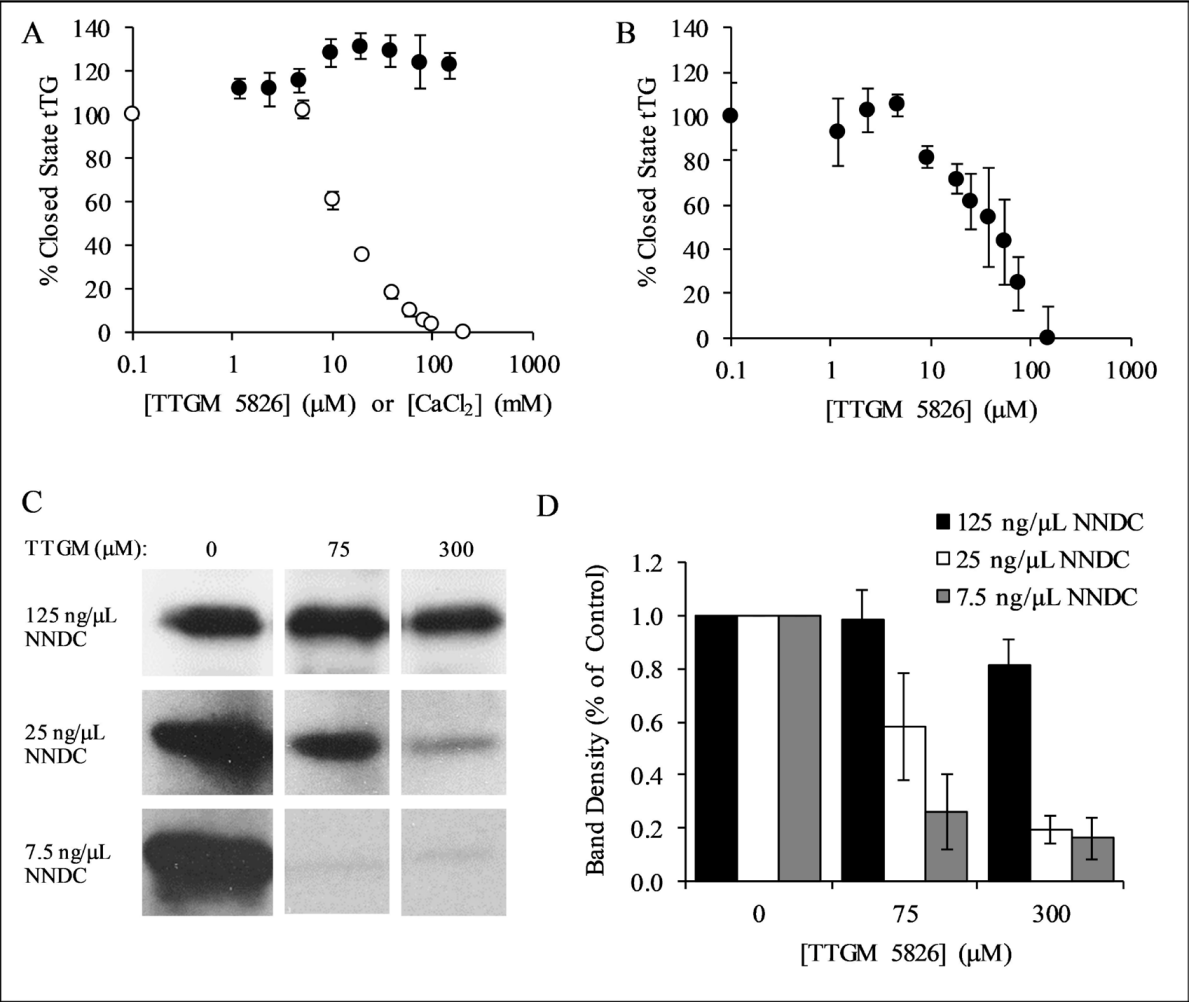
Characterization of TTGM 5826 (**A**) tTG (0.4 μM) was incubated with the indicated amounts of TTGM 5826 (black circles) or CaCl_2_ (white circles) at room temperature for 5 minutes, after which bodipy-labeled GTPγS was added to the reactions. The fluorescence of the bodipy label was measured (ex: 504 nm, em: 520 nm), and the percentage of closed state tTG determined. (**B**) tTG (0.4 μM) was incubated with 10 mM CaCl_2_ and the indicated amount of TTGM 5826 for 5 minutes, after which bodipy-labeled GTPγS and 20 mM EDTA were added. The reaction was incubated for an additional 10 minutes and then analyzed as described in (**A**). (**C**) Recombinantly expressed tTG (43 nM) was incubated at room temperature for 15 minutes with 10 mM CaCl_2_, 10 mM DTT, 62.5 μM BPA, and the indicated amounts of NNDC and of TTGM 5826. The proteins were resolved by SDS-PAGE and the extent that BPA was incorporated into NNDC was determined by probing the resulting blot with HRP-conjugated streptavidin. (**D**) Quantification of the data shown in (**C**). Band density was determined using ImageJ, and was calculated with respect to the DMSO control from each experiment. The error bars in panels (**A**), (**B**), and (**D**) represent the SD from three independent experiments. The lowest concentration data point for each series in (**A**) and (**B**) is a control experiment (no TTGM 5826 or CaCl_2_), and is assigned a low, non-zero value to allow plotting on a logarithmic axis.

We next read-out the ability of TTGM 5826 to convert tTG from the closed to the open state, by assaying the guanine nucleotide-binding capability of tTG, using the environmentally sensitive, fluorescent guanine nucleotide analog bodipy-GTP-γS. Bodipy-GTP-γS binds to and stabilizes the closed state of tTG [17]. However, the guanine nucleotide binding site of tTG is not accessible in the open state, and so incubating tTG with CaCl_2_ prevents the binding of bodipy-GTP-γS. Figure 3A shows that the addition of increasing amounts of CaCl_2_ decreases the ability of tTG to bind bodipy-GTP-γS (white circles), as monitored by changes in its fluorescence emission. However, when the same experiment was repeated using TTGM 5826 instead of CaCl_2_ (black circles), bodipy-GTP-γS binding was unaffected. These findings suggest that TTGM 5826, at sub-millimolar concentrations, is unable to induce tTG to adopt an open state conformation.

We then examined whether TTGM 5826 might function by helping to stabilize the open state conformation once it has been induced. Thus, tTG was initially incubated with CaCl_2_, to induce the open state conformation, and then TTGM 5826 was added. Ca^2+^ was chelated with EDTA, and the samples were assayed for their abilities to bind bodipy-GTP-γS. Figure 3B shows that, under these conditions, TTGM 5826 significantly reduced bodipy-GTP-γS binding, yielding an EC_50_ value of 20 μM.

We next determined whether TTGM 5826 affected the protein crosslinking activity of tTG. tTG was incubated with TTGM 5826, N,N-dimethyl casein (NNDC), and biotinylated pentylamine (BPA), which represented the amine acceptor and donor substrates of tTG, respectively, with the amount of BPA incorporated into NNDC then serving as a readout of tTG crosslinking activity. Figure 3C shows that TTGM 5826 is able to inhibit the tTG-mediated crosslinking of BPA to NNDC, and that the extent of inhibition was dependent upon the levels of NNDC being assayed. For example, even at concentrations as high as 300 μM, TTGM 5826 was unable to substantially inhibit the enzymatic activity of tTG when incubated with 125 ng/μL of NNDC (top panel). However, when 25 ng/μL of NNDC was used, 300 μM TTGM 5826 inhibited crosslinking by ~80% (middle panel), while the inhibitor was even more effective when only 7.5 ng/μL of NNDC was assayed (~80% inhibition with just 75 μM TTGM 5826, bottom panel). These findings were quantified in Figure 3D, and are consistent with TTGM 5826 acting as a competitive inhibitor (versus NNDC) of the protein crosslinking activity of tTG.

### TTGM 5826 inhibits cancer cell growth

**Figure 4:**
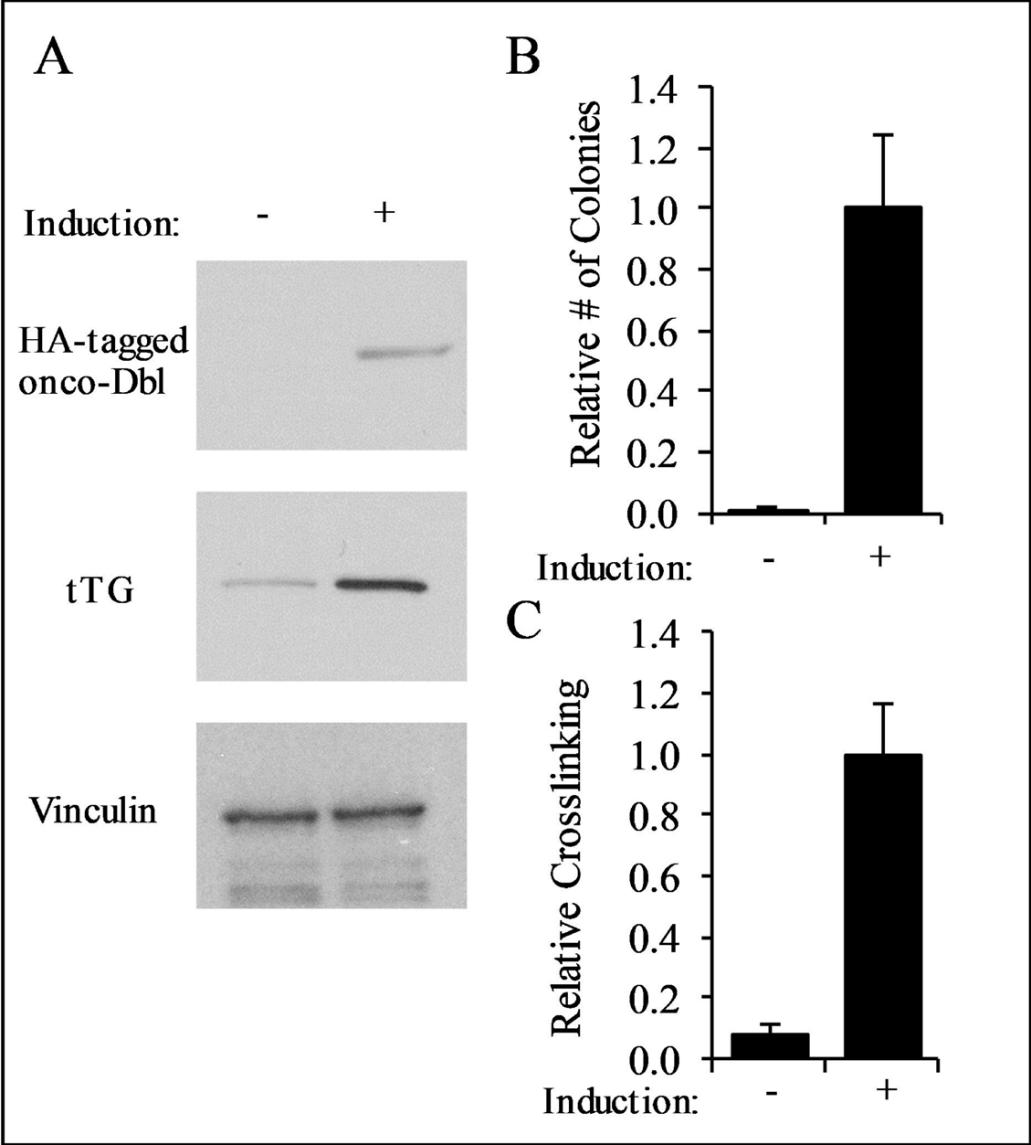
Characterization of onco-Dbl expressing MEFs (**A**) Lysates from uninduced control MEFs, and MEFs induced to express onco-Dbl by removal of doxycycline from the culturing medium, were analyzed by Western blot using HA, tTG, and vinculin (loading control) antibodies. (**B**) Soft-agar colony formation assays were performed on uninduced MEFs, and on MEFs induced to express onco-Dbl. The cells were cultured for four weeks, and the colonies formed in each condition were counted. (**C**) tTG crosslinking assays were conducted on the same cell lysates used in (**A**). An equal amount of each cell lysate was incubated with 10 mM CaCl_2_, 10 mM DTT, and 62.5 μM BPA for 15 minutes, and then resolved by SDS-PAGE. The resulting blot was probed with HRP-streptavidin, and the band densities were quantified with ImageJ. Error bars in (**B**) and (**C**) represent the SD from three independent experiments.

We next examined whether TTGM 5826 affects the growth of transformed cells. We began by using mouse embryonic fibroblasts (MEFs) that stably express an inducible form of oncogenic-Dbl (for diffuse B-cell lymphoma; onco-Dbl), a potent activator of Rho GTPases [48, 49]. MEFs cultured with doxycycline did not express onco-Dbl (Figure 4A, top panel), and thus were not transformed, as indicated by their inability to form colonies in soft agar (Figure 4B, left panel). However, upon the removal of doxycycline from the culturing media, the cells expressed onco-Dbl (Figure 4A, top panel) and exhibited anchorage-independent growth by forming large colonies in soft agar (Figure 4C), consistent with previous results [49]. The induction of onco-Dbl also resulted in a marked increase in tTG expression (Figure 4A, middle panel) and crosslinking activity, compared to non-transformed MEFs (Figure 4C).

**Figure 5:**
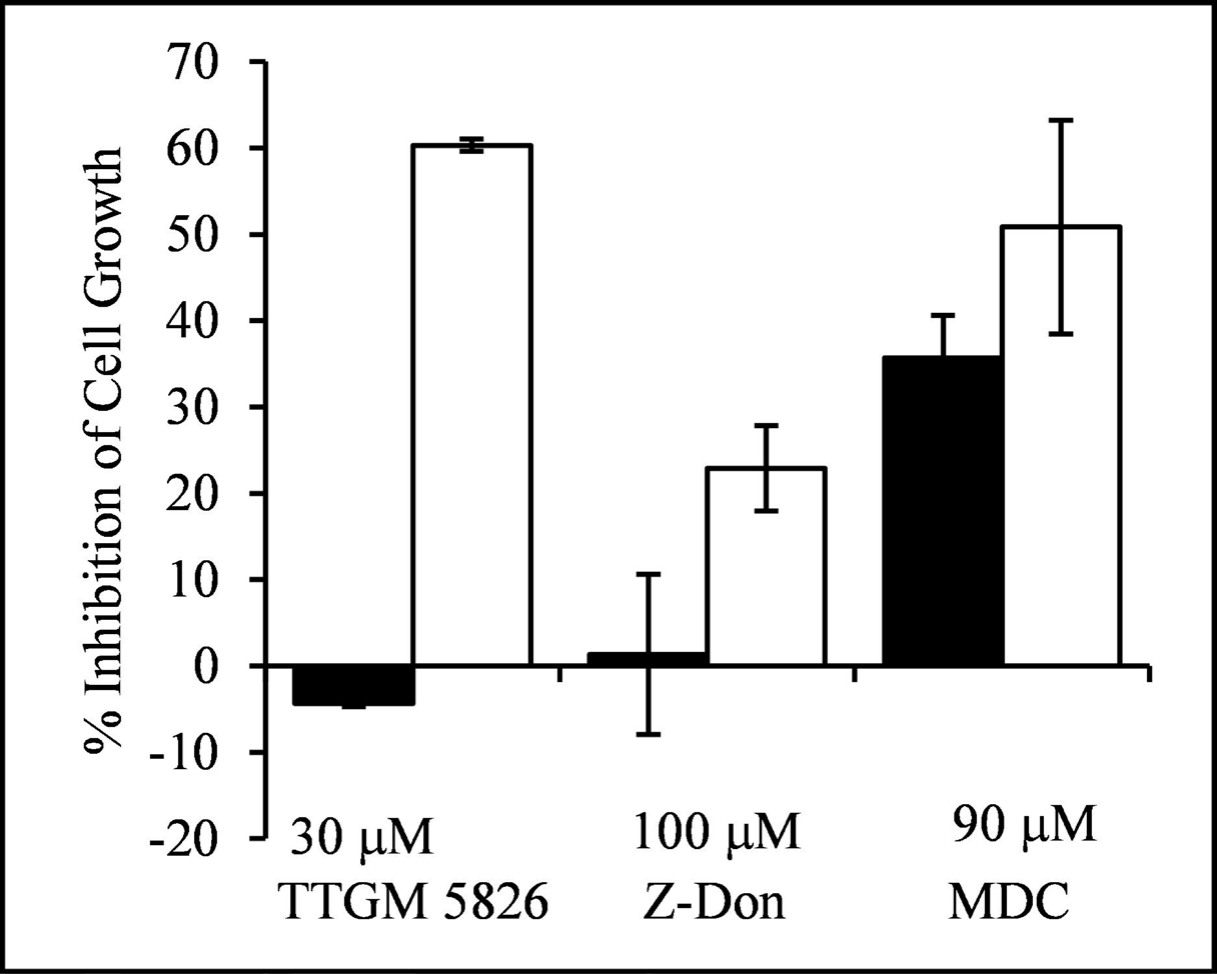
TTGM 5826 selectively inhibits the proliferation of onco-Dbl expressing MEFs Uninduced control MEFs (black), or MEFs induced to express onco-Dbl (white), were incubated for 6 days with the indicated amounts of TTGM 5826, Z-Don, or MDC. The cells were then counted, and the inhibition of cell growth, relative to DMSO treated control cells, was determined. Error bars represent the SD of three independent experiments.

Since tTG plays important roles in oncogenic transformation, this inducible model system provided us with the opportunity to compare the effects of TTGM 5826 on the growth of non-transformed versus transformed cells in a well-defined manner. Control (non-transformed) MEFs, or MEFs induced to express onco-Dbl, were treated for 6 days with either DMSO (vehicle control), TTGM 5826, or the tTG crosslinking inhibitors Z-Don or MDC. Figure 5 shows that TTGM 5826 (30 μM) exhibited a much more potent inhibition of the growth of transformed MEFs (white bar), compared to control MEFs (black bar). The conventional tTG inhibitors Z-Don (100 μM) and MDC (90 μM) also inhibited the growth of the transformed MEFs (white bars), although the differences between the non-transformed versus onco-Dbl-transformed cells were not as great when these conventional tTG inhibitors were examined.

**Figure 6:**
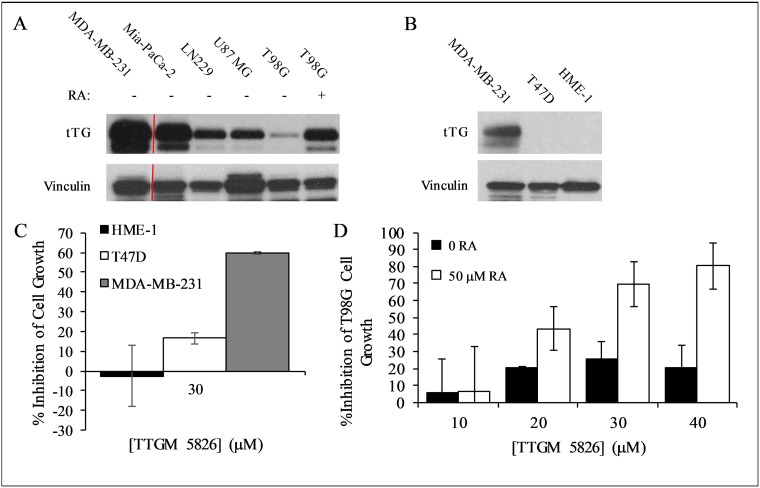
Expression levels of tTG in cancer cells and the effect of TTGM 5826 upon increasing tTG expression (**A**) Western blot analysis, using tTG and vinculin (loading control) antibodies, was performed on various cancer cell lines treated without or with RA, as indicated. (**B**) Western blot analysis, using tTG and vinculin (loading control) antibodies, was performed on the indicated cell lines. (**C**) HME-1 (black), T47D (white), and MDA-MB-231 (gray) cells were split into 12-well dishes, and then treated with 30 μM TTGM 5826 for 6 days. The cells were counted for each condition, and the percent growth inhibition was determined relative to the DMSO treated control. (**D**) T98G cells incubated for 48 hours with 0 (black) or 50 (white) μM RA were split at a low density into 12-well dishes and cultured for 6 days in the presence of the indicated amounts of TTGM 5826. The cells were counted for each condition, and the percent growth inhibition was determined relative to the DMSO treated control. Error bars in (**C**) and (**D**) represent the SD of three independent experiments.

We went on to examine the effects of TTGM 5826 on the growth of triple-negative MDA-MB-231 breast cancer cells. Treatment with either MDC or TTGM 5826 resulted in a significant reduction in cell growth, with TTGM 5826 having an IC_50_ that was roughly half that of MDC (Table 1). Four additional cancer cell lines were also analyzed, specifically LN229, T98G, and U-87 MG glioblastoma cells, and Mia-PaCa-2 pancreatic cancer cells. The ability of either MDC or TTGM 5826 to inhibit the growth of most of these cell lines was similar (Table 1). However, U-87 MG cells were resistant to MDC treatment, and T98G cells, which exhibit very low levels of tTG expression (Figure 6A, second to last lane), were resistant to both drugs.

**Table 1:** The effects of MDC and TTGM 5826 on the growth of several different cancer cell lines

		IC_50_ (μM)	
Cell Line	Tissue	MDC	TTGM 5826
MDA-MB-231	breast	60	26
Mia-PaCa-2	pancreas	50	30
LN 229	brain	66	24
T98G	brain	140	>50
U-87 MG	brain	100	30
GSC374	brain	83	16
GSC267	brain	18	11

The indicated cells were cultured for 6 days in the presence of 8 different concentrations of either MDC or TTGM 5826. On the final day, the cells were counted, and the IC_50_ for each drug was determined.

To further confirm that TTGM 5826 blocks the growth of cancer cells by binding tTG and causing it to adopt an open-state conformation, we treated T47D breast cancer cells and a human mammary epithelial cell line (HME-1 cells) with TTGM 5826. Both of these cell lines lacked detectable levels of tTG expression (Figure 6B), and so we expected them to be relatively insensitive to the drug. Indeed, Figure 6C shows that this is the case. The growth of T47D cells and HME-1 cells treated with 30 μm TTGM 5826, a concentration of drug that strongly blocked the growth of tTG expressing cancer cells (i.e. MDA-MB-231 cells), was largely unaffected, compared to control cells.

The effects of TTGM 5826 were not strictly correlated with tTG expression, perhaps because the relative amount of tTG in a crosslinking-active open state, rather than its total expression level, is the main factor determining the extent to which cancer cells are susceptible to this inhibitor. However, increasing the expression of tTG can enhance the susceptibility of some cancer cell types to TTGM 5826 inhibition. For example, this was the case for T98G cells, which express low basal levels of tTG and are relatively insensitive to TTGM 5826 (Figure 6A and Table 1). We previously showed that these cells became sensitized to MDC when tTG expression levels were upregulated, and so we examined whether the same would be true for TTGM 5826 [7]. T98G cells were treated with retinoic acid (RA), a well-known inducer of tTG expression [7, 36]. Compared to the cells treated with just DMSO (vehicle), cells treated with 50 μM RA exhibited increased expression of tTG (Figure 6A, compare last two lanes). These cells were then treated with or without TTGM 5826 for 6 days. Figure 6D shows that the DMSO-treated T98G cells were relatively insensitive to the drug, as a maximum growth inhibition of 20-30% was observed when the cells were exposed to different concentrations of TTGM 5826. However, the growth of cells treated with 50 μM RA was inhibited by TTGM 5826 in a dose dependent manner, with an IC_50_ similar to the other cancer cell lines examined, and a maximum observed growth inhibition of 80% when treated with 40 μM TTGM 5826.

### TTGM 5826 inhibits other common phenotypes of transformed cells

**Figure 7:**
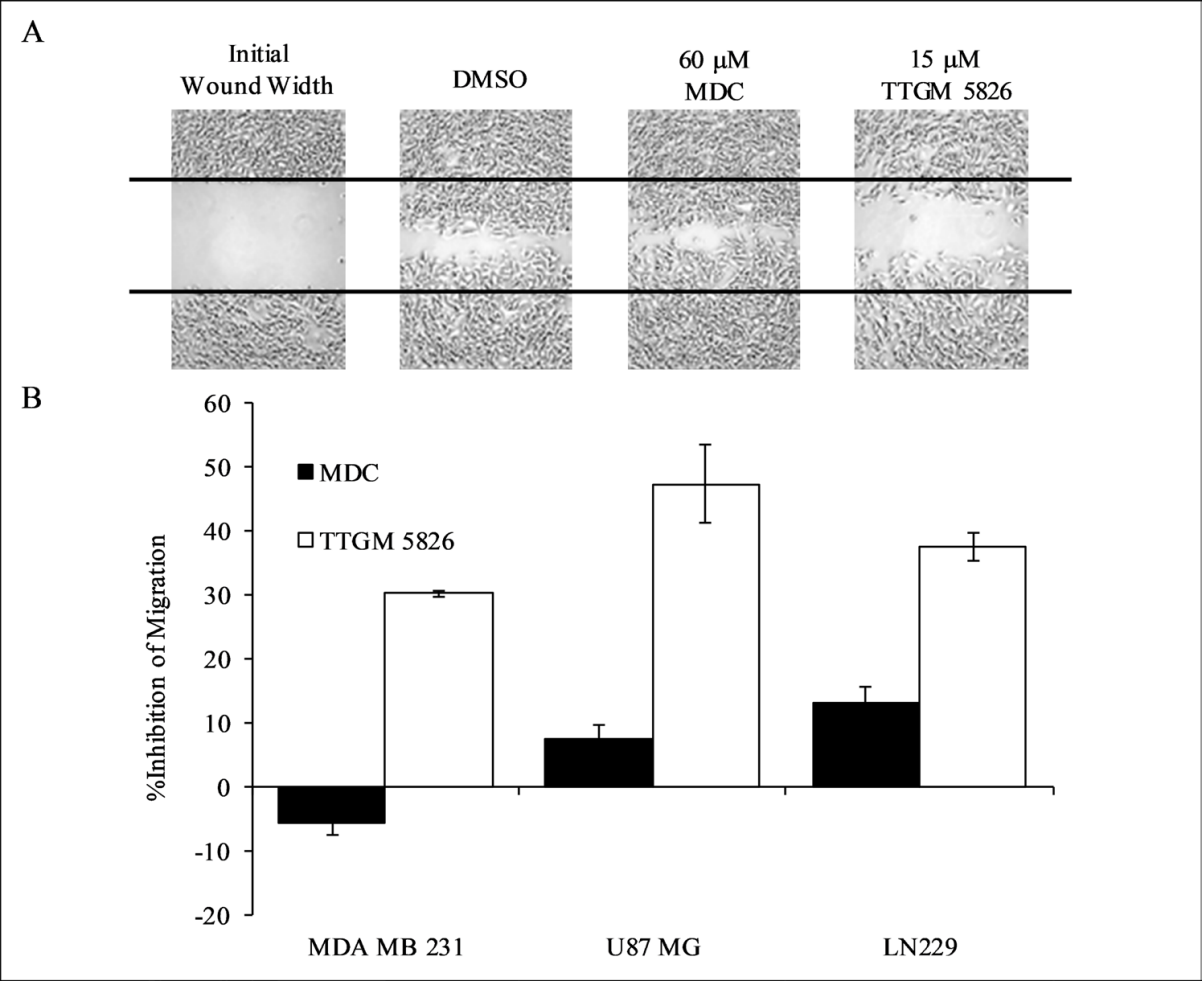
Cancer cell migration is inhibited by TTGM 5826 (**A**) Wounds were struck through confluent monolayers of MDA-MB-231 cells treated with DMSO, 60 μM MDC, or 15 μM TTGM 5826. Twelve hours later the cells were fixed and photographed to show the extent of wound closure for each condition. Representative images of the experiments are shown. The black lines trace the width of the original wound. (**B**) Quantification of the migration of the indicated cell lines treated with MDC (60 μM, black) or TTGM 5826 (15 μM, white), compared to a DMSO control. The extent of wound closure for each was determined using ImageJ. Error bars represent the SD from three independent experiments.

tTG has been found to contribute to several different transformed phenotypes in addition to cellular proliferation, including enhanced cell migration, loss of contact inhibition, and survival [4–6, 50–53]. Thus, we tested the ability of TTGM 5826 to inhibit these different transformed activities, beginning with cell migration. As shown in Figure 7A and 7B, TTGM 5826 was able to significantly slow the migration of MDA-MB-231 triple-negative breast cancer cells, as read-out by wound healing assays, under conditions where MDC showed little effect. Similar results were obtained when assaying the ability of TTGM 5826 to block the migration of the U-87 MG and LN229 brain tumor cell lines (Figure 7B). MDC had little effect against U-87 MG or LN229 cell migration.

**Figure 8:**
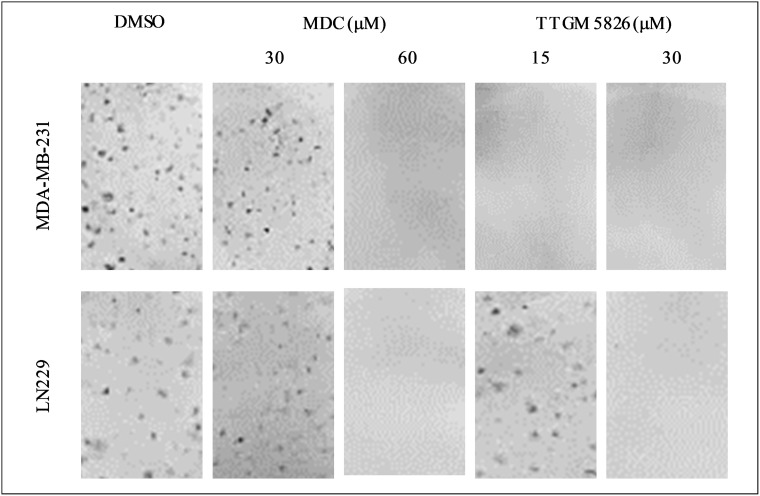
Clonogenic focus formation is inhibited by TTGM 5826 The indicated cells were plated at low density and exposed to the indicated amounts of MDC or TTGM 5826 for 10 days. The cells were then stained with crystal violet and photographed. Representative images of each condition are shown. The experiment was performed three different times.

We then conducted focus-forming assays, which reflects the ability of transformed cells and cancer cells to exhibit a loss of contact inhibition. We first tested MDA-MB-231 cells and found that they were unable to form foci when treated with as little as 15 μM TTGM 5826 (Figure 8, top panel), whereas under the same conditions, high doses (i.e. 60 μM) of MDC were needed to see the same effect. We then performed similar assays on LN229 brain cancer cells and found that the ability of this cell line to form foci was highly sensitive to TTGM 5826 (Figure 8, bottom panels).

**Figure 9:**
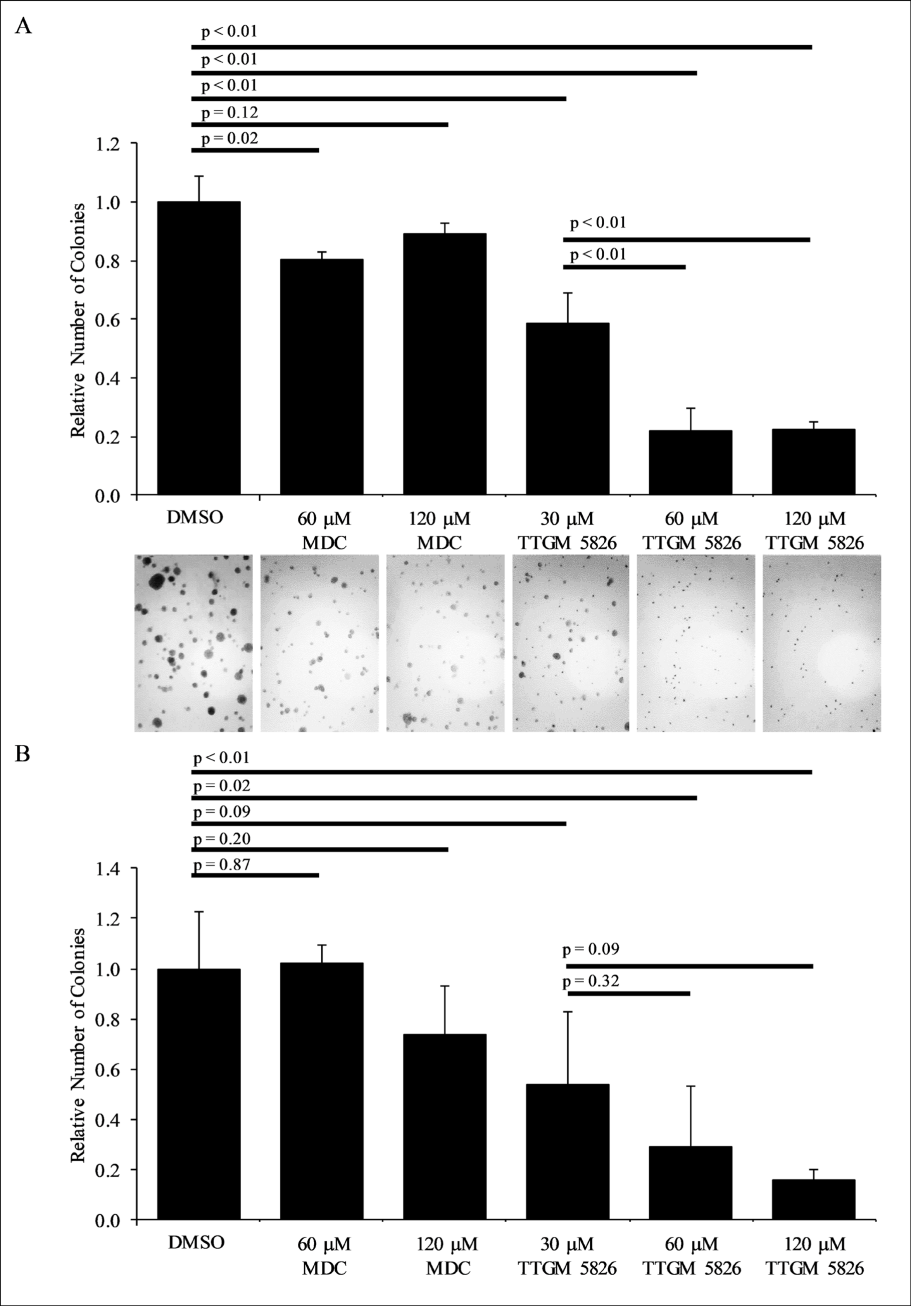
TTGM 5826 inhibits colony formation in soft agar (**A**) LN229 or (**B**) U-87 MG cells were grown in soft agar for 2 weeks, with the indicated amount of either MDC or TTGM 5826. The resulting colonies that formed for each condition were photographed (only representative images of the LN229 cells are shown) and counted. Error bars represent the SD from three separate experiments. *P*-values were calculated using a two-tailed Student's *T*-test.

Finally, we examined whether TTGM 5826 was capable of inhibiting the ability of U-87 MG or LN229 brain cancer cells to exhibit anchorage-independent growth [54, 55]. For each of these cell lines, we found that TTGM 5826 strongly blocked their abilities to form colonies in soft agar (Figure 9A and 9B). MDC was relatively ineffective at preventing colony formation under these conditions for either cell line.

### TTGM 5826 synergizes with standard of care drugs

**Figure 10:**
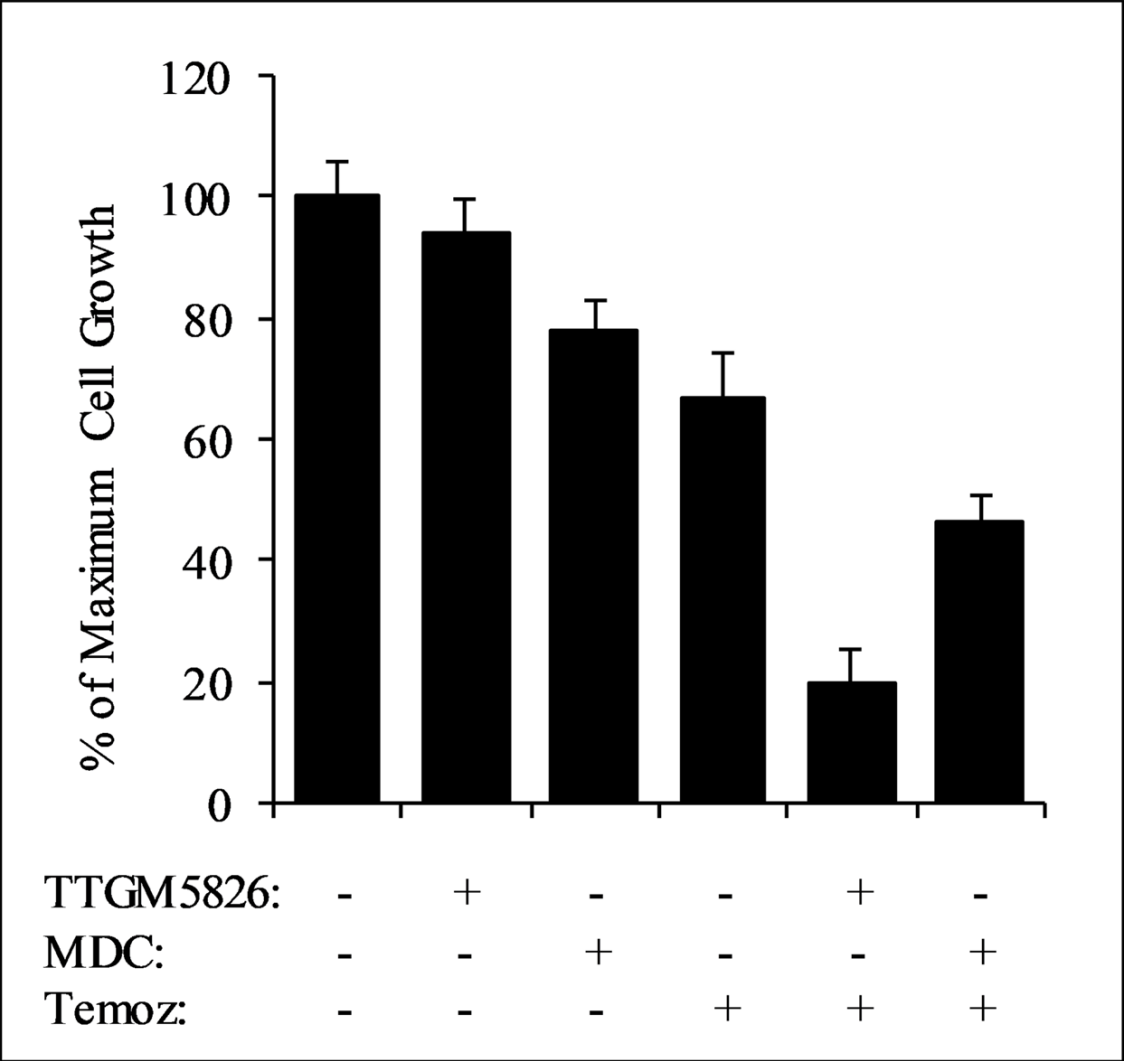
TTGM 5826 synergizes with temozolomide to inhibit the growth of U-87 MG cells U-87 MG cells were treated with various combinations of either 15 μm TTGM 5826 (TTGM) or 50 μm MDC and 15.5 μm temozolomide (Temoz) for 6 days, at which point the cells were counted. Error bars represent the SD from three different experiments.

Inhibition of tTG has been shown to greatly enhance the sensitivity of a number of different cancers to various classes of chemotherapeutic compounds [5]. Furthermore, drug cocktails are commonly used to treat cancer patients. This led us to examine whether TTGM 5826 could synergize with standard of care agents (SOCAs) for glioblastoma: temozolomide (a DNA alkylating agent), carmustine (a DNA crosslinking agent), or vincristine (a tubulin binding agent that prevents chromosome separation during metaphase). To determine drug synergy, we utilized the method developed by Chou and Talalay [7, 56]. Briefly, this method involves the calculation of a combination index (CI), which determines whether two drugs function additively (CI = 1), synergistically (CI < 1), or antagonistically (CI > 1). Cell proliferation experiments were performed to determine dose curves for U-87 MG cells treated with each of the SOCAs (IC_50_ values are reported in Table 2). We then tested each SOCA in combination with TTGM 5826 or MDC at concentrations of 1×, 0.5×, or 0.25× their relative IC_50_ values. In the U-87 MG brain cancer cell line, TTGM 5826 was synergistic with each SOCA, strongly inhibiting cell growth when using low concentrations of both inhibitors. For example, Figure 10 shows the results of experiments where U-87 MG cells were treated with different combinations of TTGM 5826, MDC, and temozolomide. When treated with 15 μM TTGM 5826, 50 μM MDC, or 15.5 μM temozolomide (half of the respective IC_50_ concentration for each inhibitor), only 10-30% inhibition of cell growth was observed (first four bars). However, when TTGM 5826 and temozolomide were combined, about 80% inhibition of growth was achieved (second to last bar). A smaller effect was observed upon combining MDC with temozolomide (last bar). Table 2 shows the resulting combination index values for each drug combination.

**Table 2:** The combination index (CI) determined for either TTGM 5826, or MDC, and the indicated standard of care agent in U-87 MG glioblastoma cells

		Combination Index at Fraction of IC_50_					
		TTGM 5826			MDC		
Drug	IC_50_	1X	0.5X	0.25X	1X	0.5X	0.25X
Temozolomide	31 μM	0.04	0.20	0.50	0.05	0.70	1.66
Carmustine	50 μM	0.28	0.52	0.46	0.29	0.84	1.17
Vincristine	0.4 nM	0.15	1.12	1.86	0.16	1.29	19.70

The CI is calculated at the indicated ratio of the IC_50_ of each drug, and the IC_50_ value used for each standard of care agent for each cell line is indicated.

### Glioma stem cells are sensitive to growth inhibition by TTGM 5826

**Figure 11:**
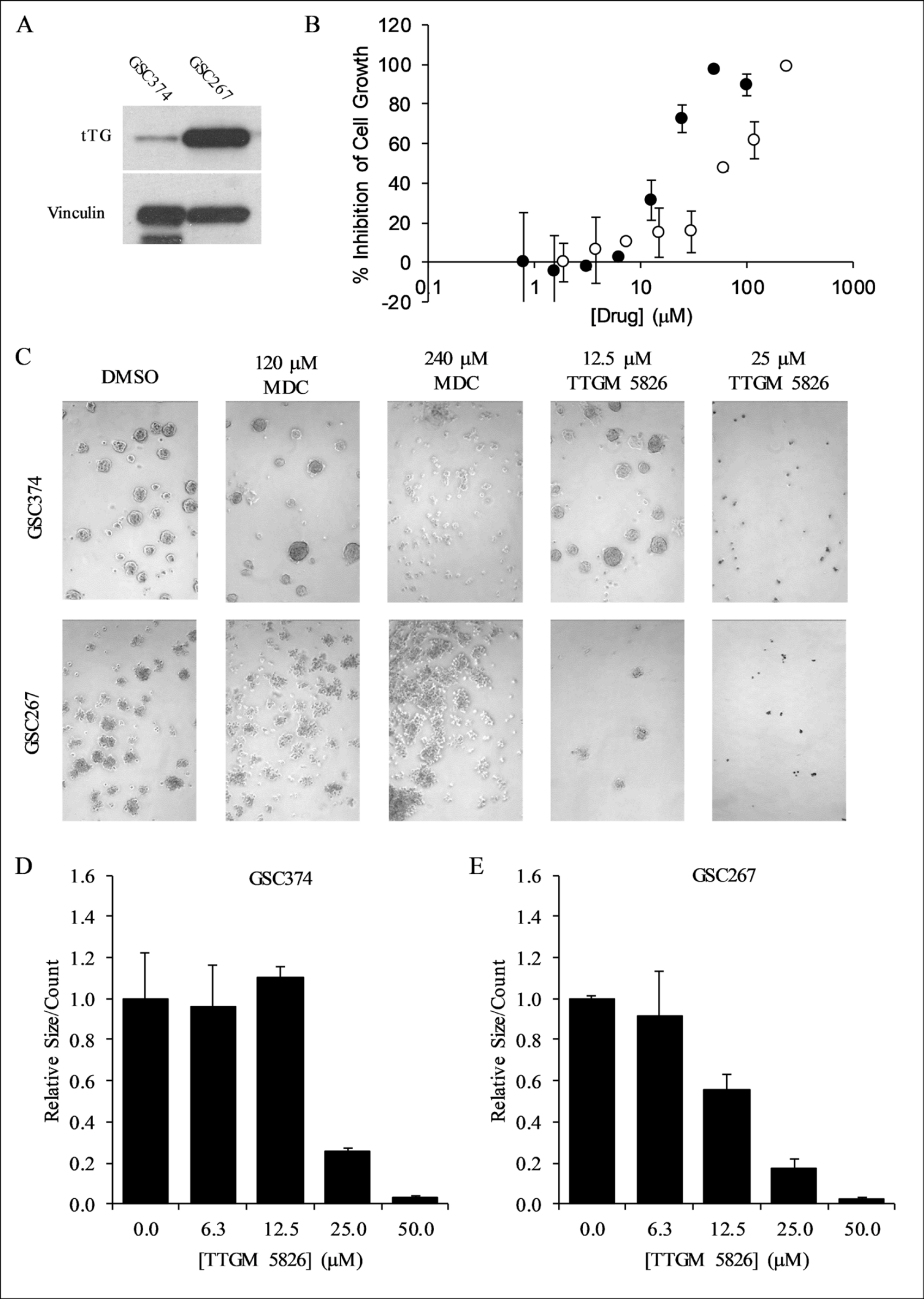
TTGM 5826 inhibits the growth and sphere forming abilities of GSCs (**A**) Western blot analysis using tTG and vinculin (loading control) antibodies was carried out on the indicated GSCs. (**B**) Growth inhibition of GSC374 cells by TTGM 5826 (black circles) or MDC (white circles). The cells were incubated with the indicated amounts of either drug for 6 days, and then counted. The percent growth inhibition was determined, relative to the DMSO control. The lowest concentration data point from either series is the DMSO control, assigned an arbitrary low value to allow plotting on a logarithmic axis. (**C**) Representative images of spheres that were formed by GSC374 or GSC267 cells treated for three days with the indicated amounts of MDC or TTGM 5826. (**D**) Average number of spheres formed by GSC374 cells treated with the indicated amounts of TTGM 5826. (**E**) Average number of spheres formed by GSC267 cells treated with the indicated amounts of TTGM 5826. Data in (**D**) and (**E**) were normalized to DMSO controls. Error bars in in (**B**), (**D**), and (**E**) represent the SD from three separate experiments.

Thus far, we have examined the effects of TTGM 5826 on various cancer cell lines, and found that it was able to inhibit multiple cancer-cell phenotypes. Since TTGM 5826 was generally more effective than the classical alternate substrate MDC in each of these cases, we became interested in determining its potential for treating GSCs. GSCs are thought to be one of the major contributing factors for the therapy resistance and tumor recurrence of glioblastoma [20, 25, 26]. They are highly resistant to radiation or pharmacological intervention, but have been shown to be sensitive to traditional tTG inhibitors such as Z-Don and MDC [25, 29–31]. Two different GSC cell lines, GSC374 and GSC267, with moderate or high levels of tTG expression, respectively (Figure 11A), were incubated with increasing amounts of TTGM 5826, MDC, or a DMSO vehicle control. Figure 11B and Table 1 show that TTGM 5826 potently inhibited the growth of these GSCs in a dose dependent manner, with IC_50_ values nearly half of those for the traditional cancer cell lines examined. Interestingly, while GSC267 cells were also highly sensitive to MDC, GSC374 cells were somewhat resistant to that drug (Figure 11B, white circles), suggesting that using TTGM 5826 to stabilize the open conformation of tTG in GSCs with only moderate amounts of tTG might be more effective than using alternate substrate inhibitors.

tTG has been shown to be important for the ability of CSCs to form spheres, a hallmark of “stemness” [5, 22, 23, 57]. As such, we tested the ability of the GSC374 and GSC267 lines to form spheres when treated with either TTGM 5826, or MDC, for 3 days. As shown in Figure 11C–11E, TTGM 5826 was able to substantially inhibit the number of spheres that formed in each of these cell lines in a dose-dependent manner. Under the same culturing conditions, MDC was far less effective at causing this effect (Figure 11C), even at concentrations well above its IC_50_.

## DISCUSSION

Through a combination of virtual screening and biochemical analysis, the small molecule TTGM 5826 was shown to stabilize the open conformation of tTG. The ability of TTGM 5826 to selectively target the tTG open state could hold clinical significance, given the high expression and activity of tTG in a number of cancers, and the cytotoxic nature of tTG when maintained in an open state conformation. TTGM 5826 was predicted during the docking simulations to bind to the crosslinking active site of tTG (Figure 2B), and indeed, we found that it exhibits a competitive inhibition versus the substrate NNDC (Figure 3C and 3D).

It seems likely that the majority of intracellular tTG in cancer cells exists in a closed state conformation, although the ability to use MDC as a tTG substrate to label tTG glutamine-donors with a fluorescent dansyl group suggests that there is at least a basal level of tTG crosslinking activity [50, 58, 59]. This would suggest that a small amount of tTG exists in the open state conformation at any given time, such that even a relatively minor perturbation to its conformational dynamics, as might be induced by TTGM 5826, could be sufficient to trigger a cytotoxic event. Indeed, when comparing the relative effectiveness of the well-known tTG inhibitor MDC, versus the newly identified TTGM 5826, at inhibiting the growth of cancer cell lines (Table 1), we found that TTGM 5826 was typically about twice as potent as MDC. The one exception was U-87 MG cells, where TTGM 5826 was able to inhibit their growth but MDC was ineffective until very high concentrations were used. Additionally, the IC_50_ values determined for TTGM 5826 in blocking the growth of the different cell lines were comparable to the EC_50_ value for stabilizing the open state conformation of recombinant tTG.

The ability of this small molecule to specifically stabilize the cytotoxic open state of the enzyme was supported by our experiments showing how TTGM 5826 affects the growth of T98G glioma cells expressing varying amounts of tTG. Specifically, we found that these cells express little tTG and are relatively insensitive to TTGM 5826. However, after treatment with RA, which significantly increased tTG expression levels, the cells became substantially more sensitive to TTGM 5826 (Figure 6D). Further support for tTG as the primary target of the small molecule comes from its ability to inhibit cell migration, clonogenic focus formation, and colony formation in soft agar, three cancer cell phenotypes in which tTG has been strongly implicated [4–6, 50–53]. It is also worth noting that the IC_50_ values for TTGM 5826 were generally under 30 μM, a concentration that strongly inhibits the growth of onco-Dbl-transformed MEFs, while having no measureable effect on non-transformed (control) MEFs. Collectively, these results are consistent with a model in which the small molecule stabilizes the open state conformation of tTG in cells, and for most cancer cell lines, the concentrations of TTGM 5826 that inhibits their growth should not effect their non-transformed counterparts. Moreover, TTGM 5826 was able to synergize with three different SOCAs (temozolomide, carmustine, and vincristine) in U-87 MG brain cancer cells to inhibit their growth.

Perhaps most exciting, we found TTGM 5826 to be effective at blocking the growth and sphere forming capabilities of two tTG-expressing GSCs, which are typically resistant to radiation and pharmacological intervention [25, 27, 28]. While TTGM 5826 and MDC were both able to inhibit the proliferation of GSCs, only TTGM 5826 significantly reduced sphere formation. These findings raise the interesting possibility that stabilizing the open state of tTG is more important than inhibiting its catalytic activity, as it pertains to GSC sphere formation. Given that sphere formation is a conserved characteristic of stem and stem-like cells, this suggests that TTGM 5826 is able to inhibit the stem-like characteristics of GSCs, which could be of value in inhibiting their unique oncogenic characteristics. Moreover, our results closely correlate with those of Kerr *et al.*, who recently showed that irreversible, peptidomimetic tTG inhibitors were able to inhibit epidermal CSCs, and proposed that this was accomplished by maintaining tTG in the open state [22]. Much like our findings, they showed that their inhibitors, alone, were ineffective at shifting tTG from a closed to an open state. However, once tTG adopted an open state, the drugs were able to bind and stabilize the open state. In contrast to our own study, however, Kerr *et al*. were able to elicit this effect only with irreversible, peptidomimetic inhibitors, which tend to have poor cell permeability [44, 46, 47].

In conclusion, we have identified a novel small molecule tTG inhibitor, TTGM 5826, that targets and stabilizes the open state conformation of tTG. TTGM 5826 is able to effectively inhibit the growth of a number of different cancer cells characterized by high tTG expression, as well as difficult to treat glioma stem cells. Furthermore, TTGM 5826 is able to strongly synergize with standard of care treatment drugs. Thus, with further development, reversible compounds which stabilize the cytotoxic open state conformation of tTG could offer new therapeutic strategies.

## MATERIALS AND METHODS

### Reagents

TTGM 5826, and other small molecules tested against tTG, were obtained from ChemBridge (San Diego, CA). GSCs were obtained as previously described [60, 61]. All other cell lines used in the study were purchased from the ATCC (Manassas, VA). RPMI 1640, DMEM, DMEM-F12, Penicillin/Streptomycin, Fungizone, B27 supplement, heparin, bFGF, EGF, Trip-LE, trypsin-EDTA, bodipy-GTP-γS, horse serum (HS), and fetal bovine serum (FBS) were purchased from Invitrogen (Waltham, MA). MEGM was obtained from Lonza (Allendale, NJ). Recombinant tTG was prepared as previously described [51]. HRP-conjugated streptavidin and BPA were obtained from Pierce Biotechnology (Waltham, MA), and the human-specific tTG antibody (MS-300-P, used for human-derived cancer cells) was from Neomarkers (Fremont, CA). The mouse-specific TG antibody (A033, used for MEFs) and Z-Don were from Zedira GMBH (Darmstadt, Germany). The anti-rabbit IgG-HRP (7074S), anti-mouse IgG-HRP (7076S), and HA antibodies (3724S) were from Cell Signaling (Danvers, MA). The vinculin antibody (V9131), purified trypsin, soybean trypsin inhibitor, temozolomide, vincristine, carmustine, MDC, DMSO, and NNDC were obtained from Sigma Aldrich (St. Louis, MO).

### Virtual screening

Libraries of commercially available compounds from ChemBridge (San Diego, CA) were downloaded from the ZINC database. The tTG crystal structure 2Q3Z was prepared using Autodock Tools [62]. The compounds (~30,000 in total) were docked to tTG using Autodock Vina [63]. The top scoring compounds were manually analyzed to eliminate molecules with common pan-assay interference (PAINS) functionalities, and the most promising remaining molecules were obtained for screening (Supplementary Figure 1) [64]. All molecules were dissolved in DMSO prior to further analysis.

### tTG crosslinking assays

Recombinantly expressed tTG (43 nM) was combined with the indicated amounts of the different drugs, or DMSO (as a control), for 5 minutes at room temperature in reaction buffer (10 mM Tris, 100 mM NaCl, pH 7.4). The crosslinking reaction was initiated by the addition of 10 mM DTT, 10 mM CaCl_2_, 62.5 μM biotinylated pentylamine (BPA), and the indicated amount of N,N-dimethyl casein (NNDC). In some cases, whole cell lysates (15 μg total protein) were used instead of recombinant tTG and NNDC in the assay. The reactions were incubated for 15 minutes at room temperature, at which point Laemmli buffer was added. The samples were boiled for 5 minutes, and then resolved by SDS-PAGE. The proteins were transferred to PVDF membranes, blocked with 10% bovine serum albumin (BSA) in BBST (20 mM sodium tetraborate, 100 mM boric acid, 80 mM sodium chloride, and 1.5% Tween-20, pH 8.5) at 4° C overnight, and probed with 1:4000 HRP-conjugated streptavidin and 5% BSA in BBST.

### Trypsin digestion assays

Recombinant tTG (2.1 μM) was combined with the indicated amounts of drug or CaCl_2_ in 10 mM Tris, 100 mM NaCl, pH 7.4, for 5 minutes, followed by the addition of trypsin to a final concentration of 2 μg/mL. The reaction was incubated for 3 hours on ice, and then 2.5 μg/mL of soybean trypsin inhibitor was added. Laemmli buffer was immediately added to each reaction, and the samples were boiled for 5 minutes and resolved by SDS-PAGE. The gels were stained with Coomassie blue to visualize the proteins.

### Bodipy-GTP binding assays

Recombinant tTG (0.4 μM) was incubated with the indicated amounts of TTGM 5826, GTP, or CaCl_2_ in reaction buffer (10 mM Tris, 100 mM NaCl, pH 7.5) at room temperature for 5 minutes. Bodipy-GTP-γS (0.5 μM final concentration) was then added to each sample, and the reactions were incubated at room temperature for 10 minutes. In some cases, samples were incubated with 10 mM CaCl_2_, along with the indicated amounts of drug, and then following the 5 minute incubation, 20 mM EDTA was added to each sample along with bodipy-GTP-γS. The fluorescence emission of each sample was measured (excitation = 504 nm; emission = 520 nm) using a 5 nm bandpass.

### Cell growth assays

The indicated cell lines were maintained at 37° C, 5% CO_2_, in RPMI 1640 supplemented with 10% FBS (for MDA-MB-231, T47D, and U-87 MG cells), DMEM supplemented with 10% FBS (for T98G, LN229, and MEFs), DMEM supplemented with 10% FBS and 2.5% HS (for Mia-PaCa-2 cells), MEGM (HME-1 cells), or DMEM-F12 plus Glutamax supplemented with 2% B27 supplement, 2.5 μg/mL heparin, 20 ng/mL bFGF, and 20 ng/mL EGF (for GSCs).

For each adherent cell line assayed, 2 × 10^4^ cells were plated in each well of a 12-well dish. Twenty-four hours later, the cells were treated with culturing media containing the indicated amounts of drug, or DMSO. The media on the cells was exchanged every 2 days, and after 6 days of growth, the cells were counted using a hemocytometer. Percent inhibition of cell growth was determined for each drug concentration relative to the DMSO control, which was considered as 100% cell growth. Dose curves were calculated in Sigma Plot 11.0, using the built-in 4 parameter logistic function. All experiments were performed in triplicate.

For the GSCs, 1 × 10^4^ cells were plated in each well of a 12-well dish. Culturing medium containing the indicated amounts of drugs, or DMSO, were added to these cultures every other day for 6 days, and the spheres that formed for each condition were either counted, or dissociated and then counted.

### Focus formation assays

Cells were plated in each well of a 6-well dish at a density of 1 × 10^3^ cells per well. The following day, the medium was replaced with fresh culturing medium containing the indicated concentration of drug. The medium on the cells was replaced every 3 days, and after 15 days of growth, the cells were fixed with 3.7% formaldehyde. The fixed cells were stained with 0.4% crystal violet in methanol, followed by extensive washing with water to remove background staining.

### Soft agar assays

Cells (1 × 10^3^ cells per well for U-87 MG cells; 5 × 10^3^ cells per well for LN229 or onco-Dbl MEF cells) combined with soft agar (0.3% 2-hydroxyethylagarose, 10% serum, 1× penicillin-streptomycin, and 1× anti-mycotic in RPMI-1640 or DMEM for U-87 MG or LN229 cells respectively) were plated on top of a hard layer of agar (0.6% 2-hydroxyethylagarose in the appropriate complete culturing medium). Every other day for 14 days, the cells were re-fed with soft agar medium containing the indicated amount of drug. The colonies were then counted.

### Migration assays

Cells were grown in 6-well dishes. Wounds were generated in the cells using a 200 μL pipette tip. The medium was then replaced with fresh medium containing the indicated amount of drug, or DMSO. When the DMSO-treated control cells had nearly closed the wound (generally 12–36 hours later), all of the cells cultured under the different conditions were fixed with 3.7% formaldehyde, and the wounds were photographed. The extent that the cells migrated into the wound was determined by measuring the open space remaining in each wound in ImageJ.

### Sphere formation assays

GSC cells were plated at a density of 1 × 10^3^ in 12-well dishes, and treated with drugs as described for the proliferation assays above. After 3 days of treatment, the total number of spheres in each well was determined by manual counting.

### Western blotting

Western Blot analysis was performed as previously described, using the specific antibodies detailed above [17].

### Data workup

Standard deviations (SDs) and *p*-values were calculated using Excel. Band densitometry was determined using ImageJ. For graphs in which multiple cell lines or Western blots were measured and reported with a single y-axis (e.g. % Inhibition of cell growth), all values were calculated with respect to the controls for that specific cell line or blot. Docking conformations were inspected using PyMol.

## SUPPLEMENTARY MATERIALS FIGURES AND TABLES



## Abbreviations

BPA: biotinylated pentylamine
CI: combination index
CSC: cancer stem cell
GSC: glioma stem cell
MDC: monodansylcadaverine
MEF: mouse embryonic fibroblast
NNDC: N,N dimethylcasein
SOCA: standard of care agent
tTG: tissue transglutaminase.

## References

[1] Gundemir S, Colak G, Tucholski J, Johnson GV (2012). Transglutaminase 2: a molecular Swiss army knife. Biochim Biophys Acta.

[2] Mann AP, Verma A, Sethi G, Manavathi B, Wang H, Fok JY, Kunnumakkara AB, Kumar R, Aggarwal BB, Mehta K (2006). Overexpression of tissue transglutaminase leads to constitutive activation of nuclear factor-kappaB in cancer cells: delineation of a novel pathway. Cancer Res.

[3] Martin A, De Vivo G, Gentile V (2011). Possible role of the transglutaminases in the pathogenesis of Alzheimer’s disease and other neurodegenerative diseases. Int J Alzheimers Dis.

[4] Antonyak MA, Li B, Regan AD, Feng Q, Dusaban SS, Cerione RA (2009). Tissue transglutaminase is an essential participant in the epidermal growth factor-stimulated signaling pathway leading to cancer cell migration and invasion. J Biol Chem.

[5] Katt WP, Antonyak MA, Cerione RA (2018). The diamond anniversary of tissue transglutaminase: a protein of many talents. Drug Discov Today.

[6] Boroughs LK, Antonyak MA, Johnson JL, Cerione RA (2011). A unique role for heat shock protein 70 and its binding partner tissue transglutaminase in cancer cell migration. J Biol Chem.

[7] Katt WP, Antonyak MA, Cerione RA (2015). Simultaneously targeting tissue transglutaminase and kidney type glutaminase sensitizes cancer cells to acid toxicity and offers new opportunities for therapeutic intervention. Mol Pharm.

[8] Antonyak MA, Li B, Boroughs LK, Johnson JL, Druso JE, Bryant KL, Holowka DA, Cerione RA (2011). Cancer cell-derived microvesicles induce transformation by transferring tissue transglutaminase and fibronectin to recipient cells. Proc Natl Acad Sci U S A.

[9] Antonyak MA, Wilson KF, Cerione RA (2012). R(h)oads to microvesicles. Small GTPases.

[10] Liu S, Cerione RA, Clardy J (2002). Structural basis for the guanine nucleotide-binding activity of tissue transglutaminase and its regulation of transamidation activity. Proc Natl Acad Sci U S A.

[11] Pinkas DM, Strop P, Brunger AT, Khosla C (2007). Transglutaminase 2 undergoes a large conformational change upon activation. PLoS Biol.

[12] Huang WC, Swietach P, Vaughan-Jones RD, Ansorge O, Glitsch MD (2008). Extracellular acidification elicits spatially and temporally distinct Ca2+ signals. Curr Biol.

[13] Clapham DE (2007). Calcium signaling. Cell.

[14] Cox AD, Fesik SW, Kimmelman AC, Luo J, Der CJ (2014). Drugging the undruggable RAS: mission possible?. Nat Rev Drug Discov.

[15] Datta S, Antonyak MA, Cerione RA (2007). GTP-binding-defective forms of tissue transglutaminase trigger cell death. Biochemistry.

[16] Colak G, Keillor JW, Johnson GV (2011). Cytosolic guanine nucledotide binding deficient form of transglutaminase 2 (R580a) potentiates cell death in oxygen glucose deprivation. PLoS One.

[17] Zhang J, Antonyak MA, Singh G, Cerione RA (2013). A mechanism for the upregulation of EGF receptor levels in glioblastomas. Cell Reports.

[18] Boroughs LK, Antonyak MA, Cerione RA (2014). A novel mechanism by which tissue transglutaminase activates signaling events that promote cell survival. J Biol Chem.

[19] Budillon A, Carbone C, Di Gennaro E (2013). Tissue transglutaminase: a new target to reverse cancer drug resistance. Amino Acids.

[20] Nassar D, Blanpain C (2016). Cancer Stem Cells: Basic Concepts and Therapeutic Implications. Annu Rev Pathol.

[21] Reya T, Morrison SJ, Clarke MF, Weissman IL (2001). Stem cells, cancer, and cancer stem cells. Nature.

[22] Kerr C, Szmacinski H, Fisher ML, Nance B, Lakowicz JR, Akbar A, Keillor JW, Lok Wong T, Godoy-Ruiz R, Toth EA, Weber DJ, Eckert RL (2017). Transamidase site-targeted agents alter the conformation of the transglutaminase cancer stem cell survival protein to reduce GTP binding activity and cancer stem cell survival. Oncogene.

[23] Cao L, Shao M, Schilder J, Guise T, Mohammad KS, Matei D (2012). Tissue transglutaminase links TGF-β, epithelial to mesenchymal transition and a stem cell phenotype in ovarian cancer. Oncogene.

[24] Kumar A, Gao H, Xu J, Reuben J, Yu D, Mehta K (2011). Evidence that aberrant expression of tissue transglutaminase promotes stem cell characteristics in mammary epithelial cells. PLoS One.

[25] Sullivan KE, Rojas K, Cerione RA, Nakano I, Wilson KF (2016). The stem cell/cancer stem cell marker ALDH1A3 regulates the expression of the survival factor tissue transglutaminase, in mesenchymal glioma stem cells. Oncotarget.

[26] Fisher ML, Keillor JW, Xu W, Eckert RL, Kerr C (2015). Transglutaminase Is Required for Epidermal Squamous Cell Carcinoma Stem Cell Survival. Mol Cancer Res.

[27] Beck B, Blanpain C (2013). Unravelling cancer stem cell potential. Nat Rev Cancer.

[28] Mao P, Joshi K, Li J, Kim SH, Li P, Santana-Santos L, Luthra S, Chandran UR, Benos PV, Smith L, Wang M, Hu B, Cheng SY (2013). Mesenchymal glioma stem cells are maintained by activated glycolytic metabolism involving aldehyde dehydrogenase 1A3. Proc Natl Acad Sci U S A.

[29] Fu J, Yang QY, Sai K, Chen FR, Pang JC, Ng HK, Kwan AL, Chen ZP (2013). TGM2 inhibition attenuates ID1 expression in CD44-high glioma-initiating cells. Neuro Oncol.

[30] Eckert RL, Fisher ML, Grun D, Adhikary G, Xu W, Kerr C (2015). Transglutaminase is a tumor cell and cancer stem cell survival factor. Mol Carcinog.

[31] Kumar A, Xu J, Brady S, Gao H, Yu D, Reuben J, Mehta K (2010). Tissue transglutaminase promotes drug resistance and invasion by inducing mesenchymal transition in mammary epithelial cells. PLoS One.

[32] Nanda N, Iismaa SE, Owens WA, Husain A, Mackay F, Graham RM (2001). Targeted inactivation of Gh/tissue transglutaminase II. J Biol Chem.

[33] Sarang Z, Tóth B, Balajthy Z, Köröskényi K, Garabuczi E, Fésüs L, Szondy Z (2009). Some lessons from the tissue transglutaminase knockout mouse. Amino Acids.

[34] Keillor JW, Apperley KY (2016). Transglutaminase inhibitors: a patent review. Expert Opin Ther Pat.

[35] Keillor JW, Apperley KY, Akbar A (2015). Inhibitors of tissue transglutaminase. Trends Pharmacol Sci.

[36] Antonyak MA, Boehm JE, Cerione RA (2002). Phosphoinositide 3-kinase activity is required for retinoic acid-induced expression and activation of the tissue transglutaminase. J Biol Chem.

[37] Karpuj MV, Becher MW, Springer JE, Chabas D, Youssef S, Pedotti R, Mitchell D, Steinman L (2002). Prolonged survival and decreased abnormal movements in transgenic model of Huntington disease, with administration of the transglutaminase inhibitor cystamine. Nat Med.

[38] Case A, Stein RL (2007). Kinetic analysis of the interaction of tissue transglutaminase with a nonpeptidic slow-binding inhibitor. Biochemistry.

[39] Caron NS, Munsie LN, Keillor JW, Truant R (2012). Using FLIM-FRET to measure conformational changes of transglutaminase type 2 in live cells. PLoS One.

[40] Al-Jallad HF, Myneni VD, Piercy-Kotb SA, Chabot N, Mulani A, Keillor JW, Kaartinen MT (2011). Plasma membrane factor XIIIA transglutaminase activity regulates osteoblast matrix secretion and deposition by affecting microtubule dynamics. PLoS One.

[41] Schaertl S, Prime M, Wityak J, Dominguez C, Munoz-Sanjuan I, Pacifici RE, Courtney S, Scheel A, Macdonald D (2010). A profiling platform for the characterization of transglutaminase 2 (TG2) inhibitors. J Biomol Screen.

[42] Griffin M, Casadio R, Bergamini CM (2002). Transglutaminases: nature’s biological glues. Biochem J.

[43] Barsigian C, Stern AM, Martinez J (1991). Tissue (type II) transglutaminase covalently incorporates itself, fibrinogen, or fibronectin into high molecular weight complexes on the extracellular surface of isolated hepatocytes. Use of 2-[(2-oxopropyl)thio] imidazolium derivatives as cellular transglutaminase inactivators. J Biol Chem.

[44] McConoughey SJ, Basso M, Niatsetskaya ZV, Sleiman SF, Smirnova NA, Langley BC, Mahishi L, Cooper AJ, Antonyak MA, Cerione RA, Li B, Starkov A, Chaturvedi RK (2010). Inhibition of transglutaminase 2 mitigates transcriptional dysregulation in models of Huntington disease. EMBO Mol Med.

[45] Yuan L, Choi K, Khosla C, Zheng X, Higashikubo R, Chicoine MR, Rich KM (2005). Tissue transglutaminase 2 inhibition promotes cell death and chemosensitivity in glioblastomas. Mol Cancer Ther.

[46] Song M, Hwang H, Im CY, Kim SY (2017). Recent Progress in the Development of Transglutaminase 2 (TGase2) Inhibitors. J Med Chem.

[47] Johnson DS, Weerapana E, Cravatt BF (2010). Strategies for discovering and derisking covalent, irreversible enzyme inhibitors. Future Med Chem.

[48] Stalnecker CA, Ulrich SM, Li Y, Ramachandran S, McBrayer MK, DeBerardinis RJ, Cerione RA, Erickson JW (2015). Mechanism by which a recently discovered allosteric inhibitor blocks glutamine metabolism in transformed cells. Proc Natl Acad Sci U S A.

[49] Kreger BT, Dougherty AL, Greene KS, Cerione RA, Antonyak MA (2016). Microvesicle Cargo and Function Changes upon Induction of Cellular Transformation. J Biol Chem.

[50] Maiuri L, Luciani A, Giardino I, Raia V, Villella VR, D’Apolito M, Pettoello-Mantovani M, Guido S, Ciacci C, Cimmino M, Cexus ON, Londei M, Quaratino S (2008). Tissue transglutaminase activation modulates inflammation in cystic fibrosis via PPARgamma down-regulation. J Immunol.

[51] Datta S, Antonyak MA, Cerione RA (2006). Importance of Ca(2+)-dependent transamidation activity in the protection afforded by tissue transglutaminase against doxorubicin-induced apoptosis. Biochemistry.

[52] Akimov SS, Krylov D, Fleischman LF, Belkin AM (2000). Tissue transglutaminase is an integrin-binding adhesion coreceptor for fibronectin. J Cell Biol.

[53] Akimov SS, Belkin AM (2001). Cell surface tissue transglutaminase is involved in adhesion and migration of monocytic cells on fibronectin. Blood.

[54] Fesus L, Thomazy V, Falus A (1987). Induction and activation of tissue transglutaminase during programmed cell death. FEBS Lett.

[55] Tucholski J, Johnson GV (2002). Tissue transglutaminase differentially modulates apoptosis in a stimuli-dependent manner. J Neurochem.

[56] Chou TC, Talalay P (1984). Quantitative analysis of dose-effect relationships: the combined effects of multiple drugs or enzyme inhibitors. Adv Enzyme Regul.

[57] Fisher ML, Kerr C, Adhikary G, Grun D, Xu W, Keillor JW, Eckert RL (2016). Transglutaminase interaction with α6/β4-integrin stimulates YAP1-dependent ΔNp63α stabilization and leads to enhanced cancer stem cell survival and tumor formation. Cancer Res.

[58] Biederbick A, Kern HF, Elsässer HP (1995). Monodansylcadaverine (MDC) is a specific *in vivo* marker for autophagic vacuoles. Eur J Cell Biol.

[59] Tovar-Vidales T, Roque R, Clark AF, Wordinger RJ (2008). Tissue transglutaminase expression and activity in normal and glaucomatous human trabecular meshwork cells and tissues. Invest Ophthalmol Vis Sci.

[60] Laks DR, Crisman TJ, Shih MY, Mottahedeh J, Gao F, Sperry J, Garrett MC, Yong WH, Cloughesy TF, Liau LM, Lai A, Coppola G, Kornblum HI (2016). Large-scale assessment of the gliomasphere model system. Neuro Oncol.

[61] Bhat KP, Balasubramaniyan V, Vaillant B, Ezhilarasan R, Hummelink K, Hollingsworth F, Wani K, Heathcock L, James JD, Goodman LD, Conroy S, Long L, Lelic N (2013). Mesenchymal differentiation mediated by NF-κB promotes radiation resistance in glioblastoma. Cancer Cell.

[62] Morris GM, Huey R, Lindstrom W, Sanner MF, Belew RK, Goodsell DS, Olson AJ (2009). AutoDock4 and AutoDockTools4: Automated docking with selective receptor flexibility. J Comput Chem.

[63] Trott O, Olson AJ (2010). AutoDock Vina: improving the speed and accuracy of docking with a new scoring function, efficient optimization, and multithreading. J Comput Chem.

[64] Baell J, Walters MA (2014). Chemistry: chemical con artists foil drug discovery. Nature.

