# Supplementary material for: A small molecule regulator of tissue transglutaminase conformation inhibits the malignant phenotype of cancer cells
Source: Oncotarget. 2018 Sep 28;9(76):34379–97. doi: 10.18632/oncotarget.26193 (PMC6188150; doi:10.18632/oncotarget.26193)
Supplement: Supplementary file 1 [file oncotarget-09-34379-s001.pdf]

A small molecule regulator of tissue transglutaminase conformation inhibits the malignant phenotype of cancer cells

SUPPLEMENTARY MATERIALS

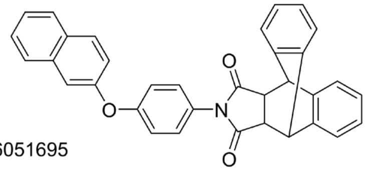

6051695

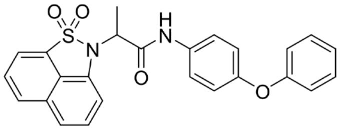

7907434

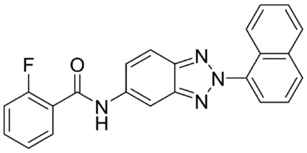

6318204

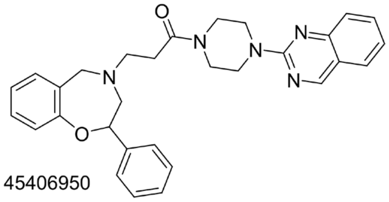

45406950

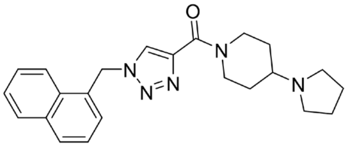

33216137

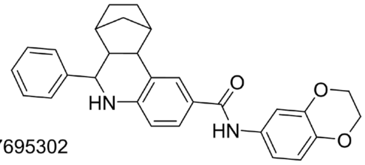

7695302

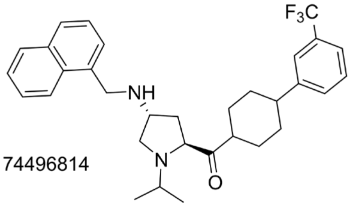

74496814

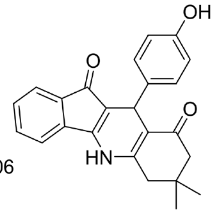

6890506

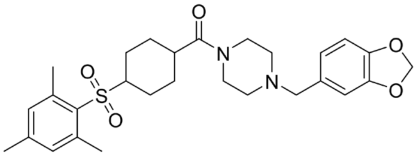

7745736

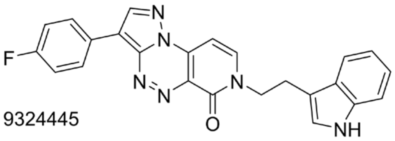

9324445

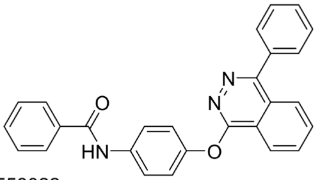

6558022

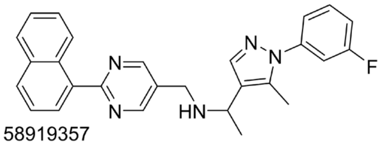

58919357

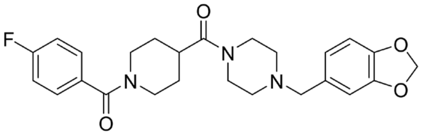

7469259

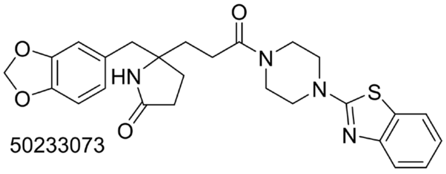

50233073

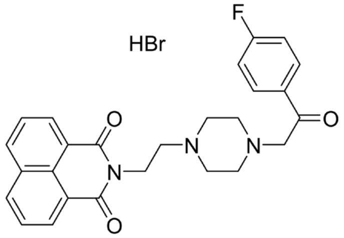

6005309

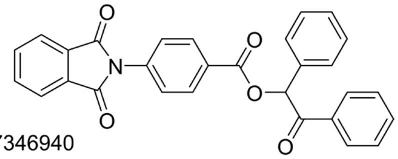

7346940

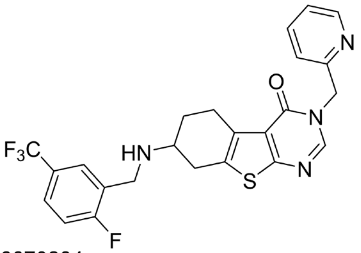

16670284

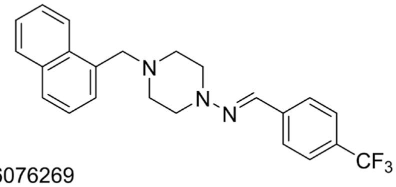

6076269

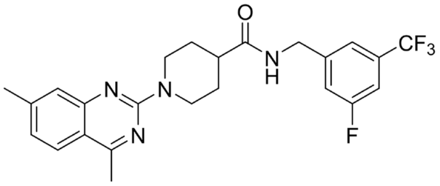

13615749

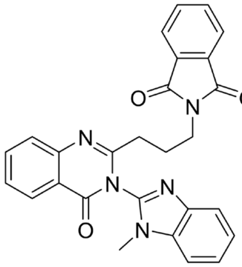

6056264

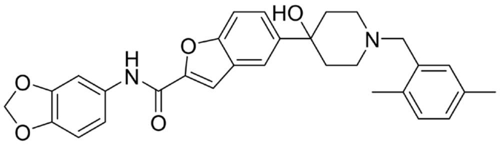

50211733

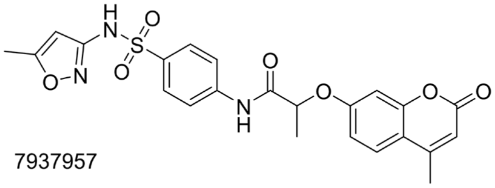

7937957

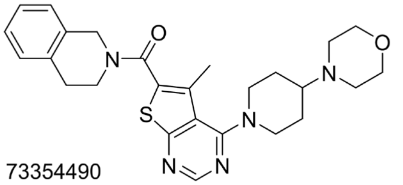

73354490

Supplementary Figure 1: The different molecules that potentially bind open state tTG. Our virtual screen identified 24 molecules that could preferentially dock onto open state tTG. The structure and corresponding ChemBridge catalog number of each molecule is shown. Compounds in the manuscript are designated TTGM #, where # is the final four digits of each compound's catalog code.
